# Supplementary material for: Preliminary Structure–Activity Relationship Study of the MMV Pathogen Box Compound MMV675968 (2,4-Diaminoquinazoline) Unveils Novel Inhibitors of Trypanosoma brucei brucei
Source: Molecules. 2022 Oct 4;27(19):6574. doi: 10.3390/molecules27196574 (PMC9571290; doi:10.3390/molecules27196574)
Supplement: Supplementary file 1 [file molecules-27-06574-s001.zip › molecules-1858445-supplementary.pdf]

Supplementary Materials

Table S1. Chemical structures of the 25 antitrypanosomal MMVPB hit compounds with known anti-kinetoplastid activity.

Compound No	MMVPB ID	IC_{50} *T.b. brucei* (µM)	*Known target parasites	Chemical Class	Chemical structure
1	MMV688180	0.002±001	*Trypanosoma brucei brucei*<br>*Trypanosoma brucei rhodesiense*<br>*Trypanosoma cruzi*	Benzenesulfonamide	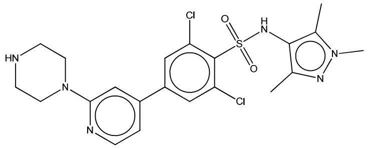
2	MMV688796	0.03±0.01	*Trypanosoma brucei brucei*<br>*Trypanosoma brucei rhodesiense*<br>*Trypanosoma cruzi*<br>*Leishmania infantum*	2,4 substituted furan	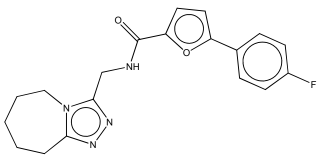

3	MMV676604	0.036±0.001	*Trypanosoma brucei brucei*<br>*Trypanosoma brucei rhodesiense*<br>*Trypanosoma cruzi*<br>*Leishmania infantum*<br>*Leishmania major*	2-aminopyramidine	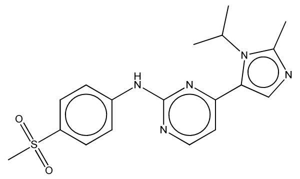
4	MMV688797	0.06±0.02	*Trypanosoma brucei brucei*<br>*Trypanosoma brucei rhodesiense*<br>*Trypanosoma cruzi*	2-aryl oxazole	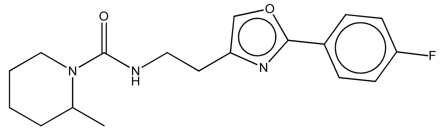
5	MMV652003	0.06±0.04	*Trypanosoma brucei brucei*<br>*Trypanosoma brucei rhodesiense*<br>*Trypanosoma cruzi*<br>*Leishmania infantum*	Benzamide	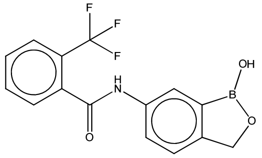

6	MMV688958	0.087±0.01	*Trypanosoma brucei brucei*<br>*Trypanosoma brucei rhodesiense*<br>*Trypanosoma cruzi*	2-aryl oxazole	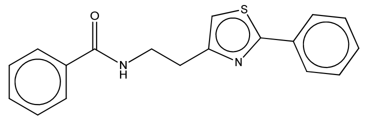
7	MMV675998	0.34±0.03	*Trypanosoma brucei brucei*<br>*Trypanosoma brucei rhodesiense*<br>*Trypanosoma cruzi*	Benzenecarboximidamide	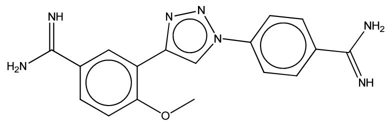
8	MMV688798	0.30±0.025	*Trypanosoma brucei brucei*<br>*Trypanosoma brucei rhodesiense*<br>*Trypanosoma cruzi*	Benzamide	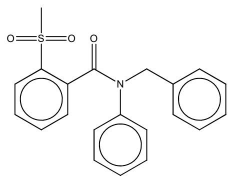
9	MMV688795	0.35±0.02	*Trypanosoma brucei brucei*<br>*Trypanosoma brucei rhodesiense*<br>*Trypanosoma cruzi*	2-aryl oxazole	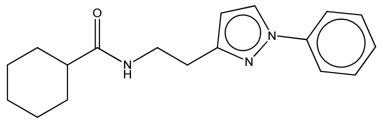

10	MMV688793	0.36±0.04	*Trypanosoma brucei brucei* *Trypanosoma brucei rhodesiense*	2-pyridyl benzamides	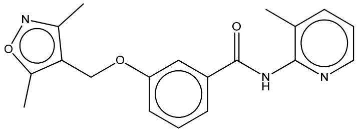
11	MMV689028	0.40±0.02	*Trypanosoma brucei brucei* *Trypanosoma brucei rhodesiense* *Trypanosoma cruzi* *Leishmania infantum*	benzyl piperazine	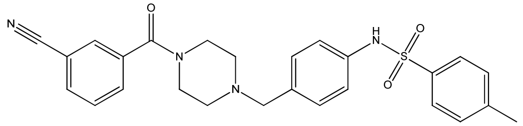
12	MMV676600	0.65±0.007	*Trypanosoma brucei brucei* *Trypanosoma brucei rhodesiense* *Trypanosoma cruzi* *Leishmania infantum*	Benzamide	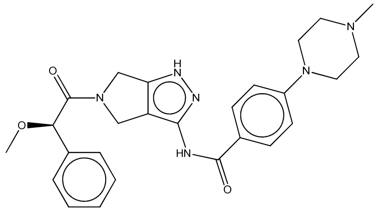
13	MMV188296	1.01±0.25	*Trypanosoma brucei brucei* *Trypanosoma brucei rhodesiense*	2-indolinecarboxamide	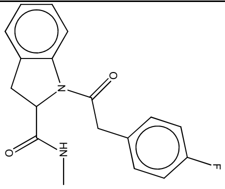

14	MMV688271	1.07±0.16	*Trypanosoma brucei brucei*<br>*Trypanosoma brucei rhodesiense*<br>*Trypanosoma cruzi*<br>*Leishmania infantum*	Guanidine	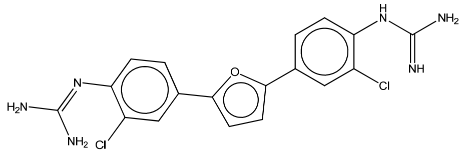
15	MMV689029	1.30±0.60	*Trypanosoma brucei brucei*<br>*Trypanosoma brucei rhodesiense*<br>*Trypanosoma cruzi*<br>*Leishmania infantum*	benzyl piperazine	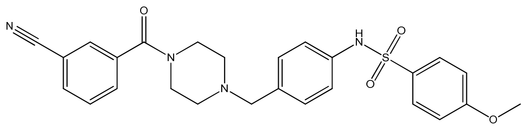
16	MMV688371	1.60±0.10	*Trypanosoma brucei brucei*<br>*Trypanosoma brucei rhodesiense*<br>*Trypanosoma cruzi*<br>*Leishmania infantum*	Benzamide	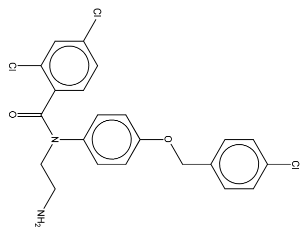

17	MMV689061	1.90±0.10	*Trypanosoma brucei brucei*<br>*Trypanosoma brucei rhodesiense*<br>*Trypanosoma cruzi*<br>*Leishmania infantum*	Acetamide	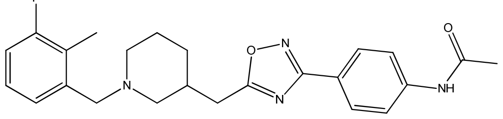
18	MMV001561	1.90±0.05	*Trypanosoma brucei brucei*<br>*Trypanosoma brucei rhodesiense*<br>*Trypanosoma cruzi*<br>*Leishmania infantum*	Propanamine	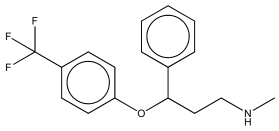
19	MMV687706	1.90±0.006	*Trypanosoma brucei brucei*<br>*Trypanosoma brucei rhodesiense*<br>*Trypanosoma cruzi*<br>*Leishmania infantum*	Piperazine	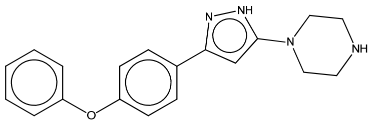

20	MMV659004	1.90±0.003	*Trypanosoma brucei brucei*<br>*Trypanosoma brucei rhodesiense*<br>*Trypanosoma cruzi*<br>*Leishmania infantum*<br>*Leishmania donovani*<br>*Leishmania major*	Pyrimidine	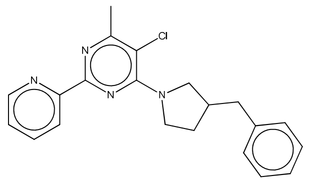
21	MMV689060	2.40±0.06	*Trypanosoma brucei brucei*<br>*Trypanosoma brucei rhodesiense*<br>*Trypanosoma cruzi*<br>*Leishmania infantum*	Piperazine	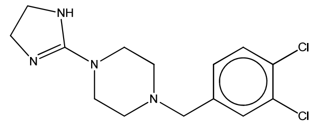
22	MMV690027	2.80±0.07	*Trypanosoma brucei brucei*	hexahydrophthalazinones	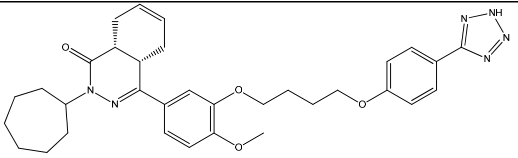

23	MMV688467	3.10±0.50	*Trypanosoma brucei brucei*<br>*Trypanosoma brucei rhodesiense*<br>*Trypanosoma cruzi*<br>*Leishmania infantum*	butyl sulfanilamide	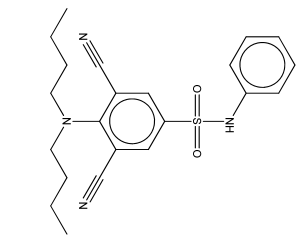
24	MMV688514	3.20±0.30	*Trypanosoma brucei brucei*<br>*Trypanosoma brucei rhodesiense*<br>*Trypanosoma cruzi*<br>*Leishmania infantum*	Benzenecarboximidamide	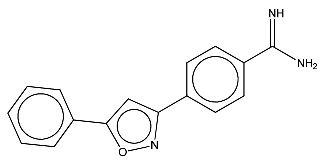
25	MMV688410	8.50±0.10	*Trypanosoma brucei brucei*<br>*Trypanosoma brucei rhodesiense*<br>*Trypanosoma cruzi*<br>*Leishmania infantum*	Acetamide	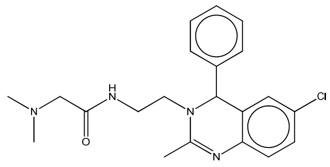 -

MMV Pathogen Box (MMVPB) compounds were tested in culture against *T. b. brucei* at serially diluted concentrations. Median inhibitory concentrations (IC_{50}) were generated from concentration–response curves using GraphPad Prism 8.0 software; MMVPB: Medicines for Malaria Venture Pathogen Box; The chemical classes of the compounds were obtained from PubChem NIH database (https://pubchem.ncbi.nlm.nih.gov); *The parasite targets for compounds were retrieved from the MMV Pathogen Box supporting information.

Table S2. Chemical structures of the 38 antitrypanosomal MMVPB hit compounds with known potency against other diseases.

Compound No	MMVPB ID	IC_{50}±SD *T.b. brucei* (µM)	*Known target disease pathogen	Chemical Class	Chemical structure
1	MMV687807	0.50±0.04	*Tubersulosis-Mycobacterium tuberculosis*	Benzamide	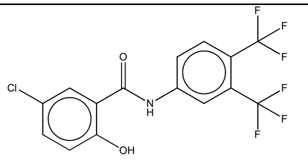
2	MMV687248	0.50±0.07	*Tubersulosis-Mycobacterium tuberculosis*	1H-Benzimidazol-2-amine	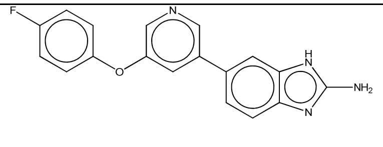
3	MMV687138	0.50±0.01	*Tubersulosis-Mycobacterium tuberculosis*	Benzamide	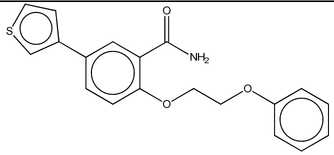
4	MMV495543	0.70±0.25	*Tubersulosis-Mycobacterium tuberculosis*	Benzamide	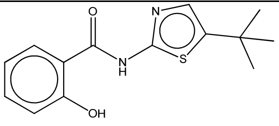
5	MMV675996	0.80±0.02	*Onchocerciasis-Onchocerca vulvulus*	cyclohexanecarboxamide	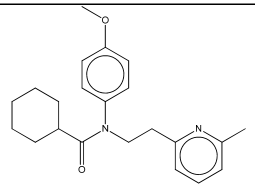

6	MMV688763	0.80±0.10	*Schistosomiasis-*<br>*Schistosoma mansoni*	pyridazinone	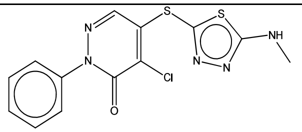
7	MMV085210	0.90±0.10	*Malaria-*<br>*Plasmodium falciparum*	Benzenesulfonamide	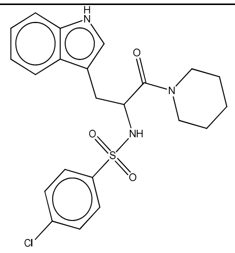
8	MMV054312	1.29±0.42	*Tubersulosis-*<br>*Mycobacterium tuberculosis*	pyrroloquinoline	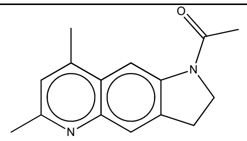
9	MMV667494	1.30±0.50	*Malaria-*<br>*Plasmodium falciparum*	Quinolone 4-<br>carboxamide	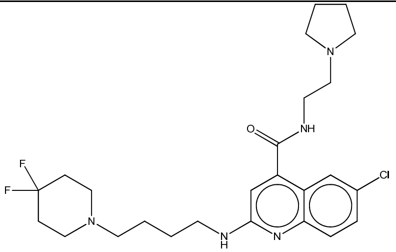

10	MMV024937	1.40±0.50	*Malaria-* *Plasmodium falciparum*	oxazolecarboxamide	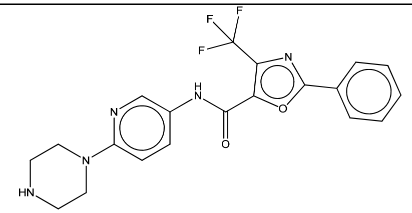
11	MMV010576	1.50±0.30	*Malaria-* *Plasmodium falciparum*	2-amino Pyridines	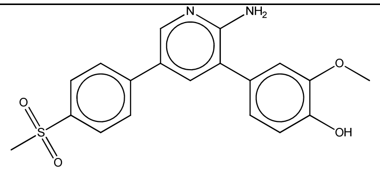
12	MMV687812	1.70±0.10	*Tubersulosis-* *Mycobacterium tuberculosis*	2-Pyrazinecarboxamide	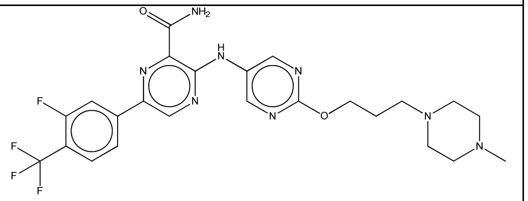
13	MMV022029	1.70±0.70	*Malaria-* *Plasmodium falciparum*	biaryl sulfonamide	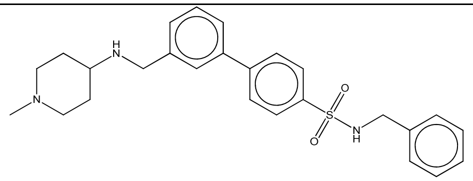

14	MMV153413	1.70±0.10	*Tubersulosis-* *Mycobacterium tuberculosis*	tetrasubstituted thiophene	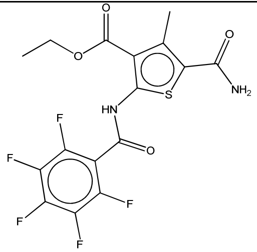
15	MMV687703	1.70±0.06	*Tubersulosis-* *Mycobacterium tuberculosis*	Benzimidazole	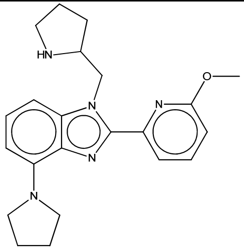
16	MMV062221	1.70±0.60	*Malaria-* *Plasmodium falciparum*	phenylpyrazolamine	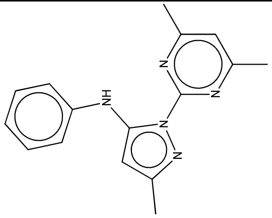
17	MMV022478	1.70±0.01	*Malaria-* *Plasmodium falciparum*	pyrazolo (1.5-a)pyrimidine	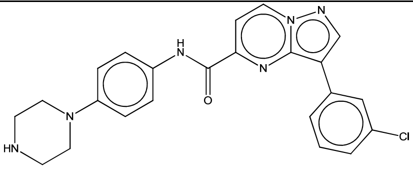

18	MMV028694	1.80±0.10	*Malaria-Plasmodium falciparum*	2.4 disubstituted pyrimidine	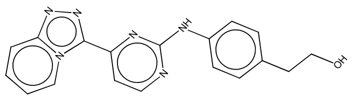
19	MMV024035	1.80±0.10	*Malaria-Plasmodium falciparum*	Thiophene carboxamide	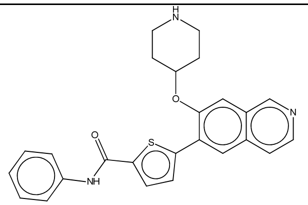
20	MMV676512	1.90±0.07	*Tubersulosis-Mycobacterium tuberculosis*	1H-Imidazole-5-carboxamide	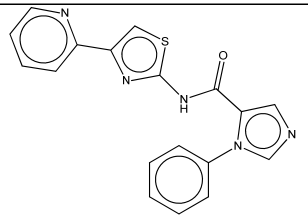
21	MMV661713	1.90±0.02	*Tubersulosis-Mycobacterium tuberculosis*	4-pyridyl-2-aryl pyrimidine	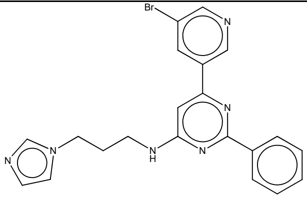

22	MMV687251	1.90±0.01	*Tubersulosis-* *Mycobacterium tuberculosis*	pyrimidine	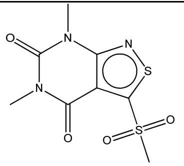
23	MMV020670	1.90±0.02	*Malaria-* *Plasmodium falciparum*	6-naphthyridine-2-carboxamide	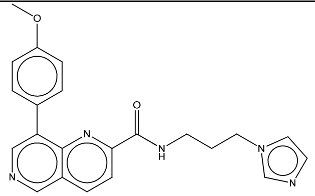
24	MMV687765	2.30±0.30	*Tubersulosis-* *Mycobacterium tuberculosis*	Pyrimidine	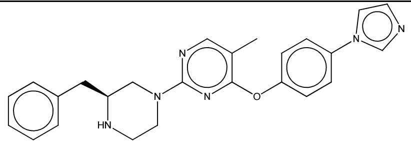
25	MMV675968	2.80±0.07	*Cryptosporidiosis-* *Cryptosporidium parvum*	aminoquinazoline	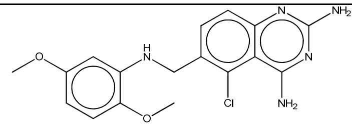
26	MMV688124	2.90±0.08	*Tubersulosis-* *Mycobacterium tuberculosis*	Benzenesulfonamide	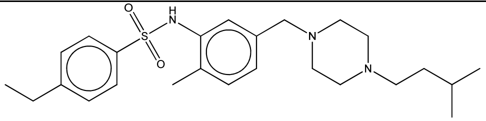
27	MMV688703	3.18±1.30	*Toxoplasmosis-* *Toxoplasma gondii*	Pyridines	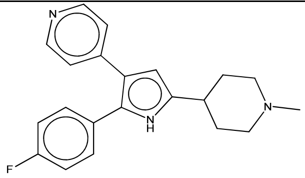

28	MMV688417	4.38±0.90	*Toxoplasmosis-* *Toxoplasma gondii*	Pyrazolo [3,4-d]pyrimidinamine	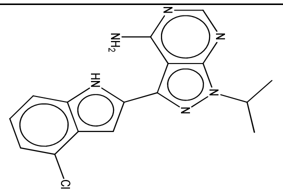
29	MMV023969	8.09±0.30	*Tubersulosis-* *Mycobacterium tuberculosis*	Isoquinoline	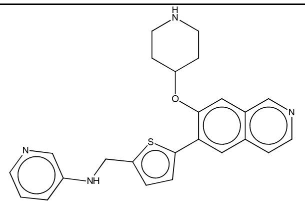
30	MMV688761	8.33±0.09	*Schistosomiasis-* *Schistosoma mansoni*	Benzamide	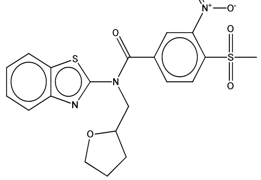
31	MMV688768	8.36±0.08	*Schistosomiasis-* *Schistosoma mansoni*	2,3 disubstituted indole	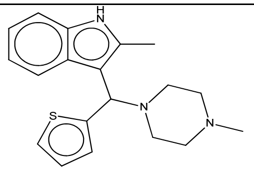
32	MMV023233	8.43±0.06	*Malaria-* *Plasmodium falciparum*	quinolineamine	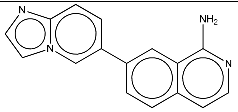

33	MMV006901	8.45±0.05	*Malaria-* *Plasmodium falciparum*	2,4-aminoquinoline	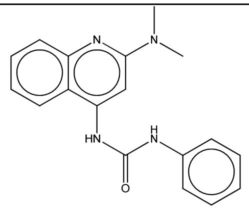
34	MMV688854	8.50±0.06	*Cryptosporidiosis-* *Cryptosporidium parvum*	pyrazolo pyrimidineamine	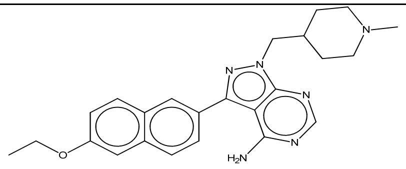
35	MMV016136	8.61±0.30	*Malaria-* *Plasmodium falciparum*	pyrazolo pyridineamine	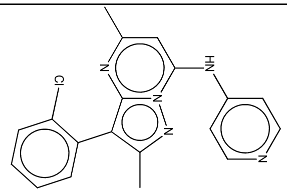
36	MMV011511	8.94±0.70	*Malaria-* *Plasmodium falciparum*	piperidineamine	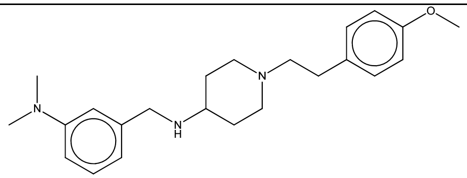
37	MMV676411	9.44±0.09	*Tuberculosis-* *Mycobacterium tuberculosis*	Propanamide	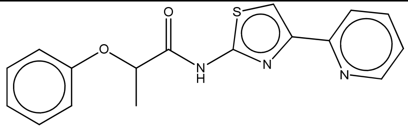

38	MMV024311	9.78±0.20	*Tuberculosis-Mycobacterium tuberculosis*	1H-Indole	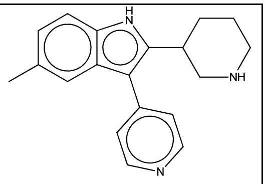

MMV Pathogen Box (MMVPB) compounds were tested in culture against *T. b. brucei* at serially diluted concentrations. Median inhibitory concentrations (IC_{50}) were generated from concentration–response curves using GraphPad Prism 8.0 software; MMVPB: Medicines for Malaria Venture Pathogen Box; The chemical classes of the compounds were obtained from PubChem NIH database (https://pubchem.ncbi.nlm.nih.gov); *The disease targets for compounds were retrieved from the MMV Pathogen Box supporting information.
